# AChR β-Subunit mRNAs Are Stabilized by HuR in a Mouse Model of Congenital Myasthenic Syndrome With Acetylcholinesterase Deficiency

**DOI:** 10.3389/fnmol.2020.568171

**Published:** 2020-12-09

**Authors:** Jennifer Karmouch, Perrine Delers, Fannie Semprez, Nouha Soyed, Julie Areias, Guy Bélanger, Aymeric Ravel-Chapuis, Alexandre Dobbertin, Bernard J. Jasmin, Claire Legay

**Affiliations:** ^1^CNRS UMR 8003, Université de Paris, Sorbonne Paris Cité, Paris, France; ^2^Department of Cellular and Molecular Medicine, Faculty of Medicine, University of Ottawa, Ottawa, ON, Canada

**Keywords:** neuromuscular junction, congenital myasthenic syndromes, p38 MAPK, HuR, acetylcholine receptor mRNA, ColQ

## Abstract

Collagen Q (COLQ) is a specific collagen that anchors acetylcholinesterase (AChE) in the synaptic cleft of the neuromuscular junction. So far, no mutation has been identified in the *ACHE* human gene but over 50 different mutations in the *COLQ* gene are causative for a congenital myasthenic syndrome (CMS) with AChE deficiency. Mice deficient for COLQ mimic most of the functional deficit observed in CMS patients. At the molecular level, a striking consequence of the absence of COLQ is an increase in the levels of acetylcholine receptor (AChR) mRNAs and proteins *in vivo* and *in vitro* in murine skeletal muscle cells. Here, we decipher the mechanisms that drive AChR mRNA upregulation in cultured muscle cells deficient for COLQ. We show that the levels of AChR β-subunit mRNAs are post-transcriptionally regulated by an increase in their stability. We demonstrate that this process results from an activation of p38 MAPK and the cytoplasmic translocation of the nuclear RNA-binding protein human antigen R (HuR) that interacts with the AU-rich element located within AChR β-subunit transcripts. This HuR/AChR transcript interaction induces AChR β-subunit mRNA stabilization and occurs at a specific stage of myogenic differentiation. In addition, pharmacological drugs that modulate p38 activity cause parallel modifications of HuR protein and AChR β-subunit levels. Thus, our study provides new insights into the signaling pathways that are regulated by ColQ-deficiency and highlights for the first time a role for HuR and p38 in mRNA stability in a model of congenital myasthenic syndrome.

## Introduction

The collagen Q (COLQ) plays a critical role at the mammalian neuromuscular junction (NMJ) through the anchoring and accumulation of acetylcholinesterase (AChE) in the synaptic basal lamina (Legay, 2018). It allows AChE to control acetylcholine level in the synaptic space thereby limiting the duration of synaptic transmission. To date, over 50 mutations have been identified in the human *COLQ* gene, all of which leading to a congenital myasthenic syndrome (CMS) with endplate AChE deficiency (Mihaylova et al., 2008; Vanhaesebrouck and Beeson, 2019). Congenital myasthenic syndrome with AChE deficiency is first detected in childhood and is characterized by muscle weakness, muscle atrophy, and slow pupillary response to light stimulation. As the disease progresses, skeletal deformities (e.g., lordosis or kyphoscoliosis), ptosis, ophthalmoplegia, and difficulty breathing can occur with a high risk of lethality (Mihaylova et al., 2008). A mouse model of CMS with AChE deficiency has been generated by the knock-out of the *ColQ* gene portraying the main characteristic features observed in patients suffering from CMS with muscle weakness and NMJ disorganization (Feng et al., 1999; McMacken et al., 2019). Furthermore, reintroduction of *COLQ* in these mutant mice has been shown to restore a normal phenotype in motor functions, synaptic transmission and structure of the NMJ (Ito et al., 2012).

Collagen Q contains a proline-rich attachment domain (PRAD) in the N-terminus that binds to a tryptophan amphiphilic tetramerization (WAT) sequence at the C-terminus of the AChE splice variant AChE_*T*_ (Massoulié and Bon, 2006). Two clusters of basic residues are found in the central collagenous domain and bind the proteoglycan perlecan (Deprez and Inestrosa, 1995; Peng et al., 1999; Arikawa-Hirasawa et al., 2002), which in turn binds to the transmembrane protein dystroglycan (Jacobson et al., 2001). This collagenous domain is followed by a trimerization domain that allows the formation of the triple helix of COLQ (Bon et al., 2003). COLQ is synthetized by skeletal muscle cells and *ColQ* mRNAs and protein accumulate at the NMJ. However, depending on the type of muscle, COLQ is also present in extrajunctionnal regions although at reduced levels (Krejci et al., 1999). COLQ/AChE accumulation at the synapse is dictated by the interaction between COLQ C-terminus and the muscle specific kinase (MuSK), a postsynaptic signaling hub which is clustered at the NMJ (Cartaud et al., 2004). Together with its co-receptor LRP4 (low-density lipoprotein receptor-related protein 4), MuSK is responsible for acetylcholine receptor (AChR) aggregation and is indispensable for the formation of the NMJ (Li et al., 2018). In COLQ-deficient muscle cells, the number of AChR clusters is upregulated as a result of an increase in AChR mRNA levels (Sigoillot et al., 2010, 2016).

One of the mechanisms regulating mRNA levels occurs through the regulation of their stability by trans-acting factors such as RNA-binding proteins (RBPs), non-coding RNAs and microRNAs. Previous studies have reported that human antigen R (HuR), a member of the ELAVL1/Hu family of RBPs, is critical during skeletal myogenesis for the fusion of myoblasts into myotubes (Van der Giessen et al., 2003; Von Roretz et al., 2011). Human antigen R is known to coordinate the expression of muscle early genes such as *Myod1*, *Myog*, and *p21* (Figueroa et al., 2003) but also to control later expressed genes by targeting synaptic mRNAs such as *Utrn* and *Ache* (Deschenes-Furry et al., 2005; Chakkalakal et al., 2008). In addition to these mRNAs, we have shown that HuR increases the stability of AChR β-subunit transcripts following skeletal muscle denervation (Joassard et al., 2015). Since COLQ-deficient mice also exhibits an increase in the levels of AChR β-subunit mRNAs as well as a number of other synaptic mRNAs (Sigoillot et al., 2016), we explore in the present study, the possibility that COLQ controls β-subunit mRNA stabilization through a mechanism mediated by HuR in a model for CMS with AChE deficiency due to the loss of COLQ.

Here, we provide novel insights into the mechanism that regulates COLQ-mediated AChR β-subunit expression. We found that: (i) in the absence of COLQ, AChR β-subunit mRNA stability and HuR levels are increased, and HuR is translocated to the cytoplasm; (ii) HuR interacts specifically with the ARE (AU-rich elements, AREs) in the 3′UTR of AChR β-subunit transcripts to positively regulate their steady-state levels; (iii) COLQ controls HuR levels through p38 activation specifically in myotubes; and (iv) modulating p38 activity induces parallel variations in HuR proteins and AChR mRNA levels. Collectively, these findings highlight a new pathway in the CMS with AChE deficiency.

## Results

### The Lack of COLQ Induces an Increase in AChR β-Subunit mRNA Stability

To explore the mechanisms of AChR mRNA upregulation in absence of COLQ, we decided to focus on AChR β-subunit mRNAs. The choice of this subunit mRNA was dictated by its constant expression during life and the crucial physiological role of the encoded subunit. Indeed this transcript encodes a key subunit of the AChR pentamer that interacts with rapsyn, a peripheral membrane protein (Wu et al., 2010). This interaction is responsible for AChR clustering at the NMJ, a process which is regulated by the phosphorylation of the AChR β-subunit (Wu et al., 2010), and is rate-limiting in dictating the amount and concentration of AChR pentamers at post-synaptic sites. In addition, we have shown previously, in another pathological context that AChR β-subunit mRNAs are post-transcriptionally regulated (Joassard et al., 2015). We thus first tested the influence of COLQ deficiency on the stability of this specific transcript (Karmouch, 2014).

**FIGURE 1:**
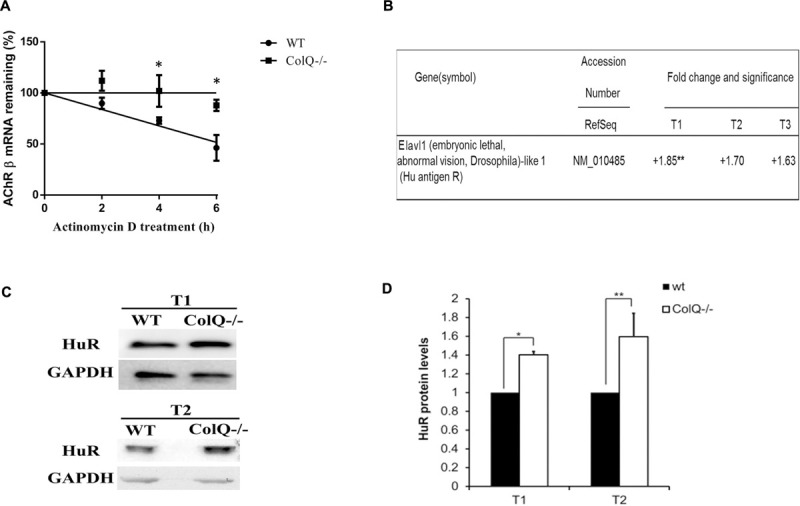
AChR β-subunit mRNA stability, HuR mRNA and protein levels are increased in COLQ-deficient myotubes compared to WT myotubes. **(A)** 4 day differentiated myotubes were treated with the transcriptional inhibitor Actinomycin D (4 μg/ml) for 2, 4, and 6 h and mRNAs were quantified by RT-QPCR relative to 18S. AChR β-subunit mRNA decay curves are shown with all values standardized to 100% RNA at time 0. Values were plotted on a semi-log scale as percentage of mRNA quantified at time 0. Values are expressed as means ± SEM (*n* = 3). *p < 0.05; **p < 0.01. **(B)** Fold changes in HuR/Elavl1 mRNAs purified from COLQ-deficient myotubes and compared to WT myotubes (*n* = 3). **(C)** Representative western blot and **(D)** Quantification of HuR in WT and COLQ-deficient myotubes at T1 and T2 time-points normalized to GAPDH. T1, T2, and T3 represent different stages of myotubes formation (T1) and further differentiation (T2 and T3). Data are the mean values of four independent experiments ± SEMs. *p < 0.05; **p < 0.01.

To investigate this mechanism, we used the WT and the COLQ-deficient polyclonal mMLCL muscle cell lines (see section “Materials and Methods” for the cell lines). Acetylcholinesterase clusters are observed in the WT cell line but not in the COLQ-deficient cell line in agreement with the role of COLQ. Two days after myotube formation, cultures were treated with 4 μg/ml of the transcriptional inhibitor actinomycin D and total RNA was extracted at different time points (0, 2, 4, and 6 h post treatment). mRNAs were isolated, reverse-transcribed (RT) and quantified by Q-PCR. Quantification at the different time points showed that AChR β-subunit mRNA levels remaining in WT and COLQ-deficient cells were significantly different after 4 and 6 h of treatment. AChR β-subunit transcripts decayed at a significantly higher rate in WT cells compared to COLQ-deficient cells indicating that AChR β-subunit mRNAs are stabilized in absence of COLQ (Figure 1A). The half life of the AChR β-subunit mRNAs in wt cells was 5.6 h whereas the half life of the AChR β-subunit mRNAs in ColQ-deficient cells was 19.2 h. Thus, increased AChR mRNA stability correlates with the previously observed increase in AChR mRNA levels in COLQ-deficient cells.

### HuR Is a Candidate Protein for AChR Transcript Stability in Absence of COLQ

The next question raised by these findings concerned the identity of the trans-acting factors that could influence the stability of AChR β-subunit mRNA. The group of Jasmin in collaboration with our group has previously shown that HUR (ELAVL1), the ubiquitously expressed RBP stabilizes AChR β-subunit mRNA in response to musle denervation by interacting with the ARE contained in its 3′UTR (Joassard et al., 2015). To explore a similar process in absence of COLQ, we initially compared the levels of HuR mRNA in WT and COLQ-deficient muscle cells. Data were obtained from a microarray previously performed at various stages of WT and COLQ-deficient myotube differentiation (time-points defined as T1, T2, and T3) (Cartaud et al., 2004; Sigoillot et al., 2016). At T1 (newly formed myotubes), myotubes are observed without yet detectable AChR clusters. T2 corresponds to further differentiated myotubes (2 days older than T1) and is characterized by the presence of AChR clusters. Three days after T2, T3 is marked by clusters of AChE-COLQ in WT cells but not in COLQ-deficient cells and appearance of the first spontaneous muscle contractions in both cell lines. In the absence of COLQ, an increase in HuR mRNA compared to WT muscle cells was observed at all time points (T1, + 1.85; T2, + 1.70; T3, + 1.63), and was significant at T1 (Figure 1B). HuR protein quantification from western blots showed a similar result in the absence of COLQ with a significant increase in HuR in two of the three stages of myotube differentiation with a 40% increase at T1 (*p* < 0.05) and a 60% increase at T2 (*p* < 0.01) (Figures 1C,D).

**FIGURE 2:**
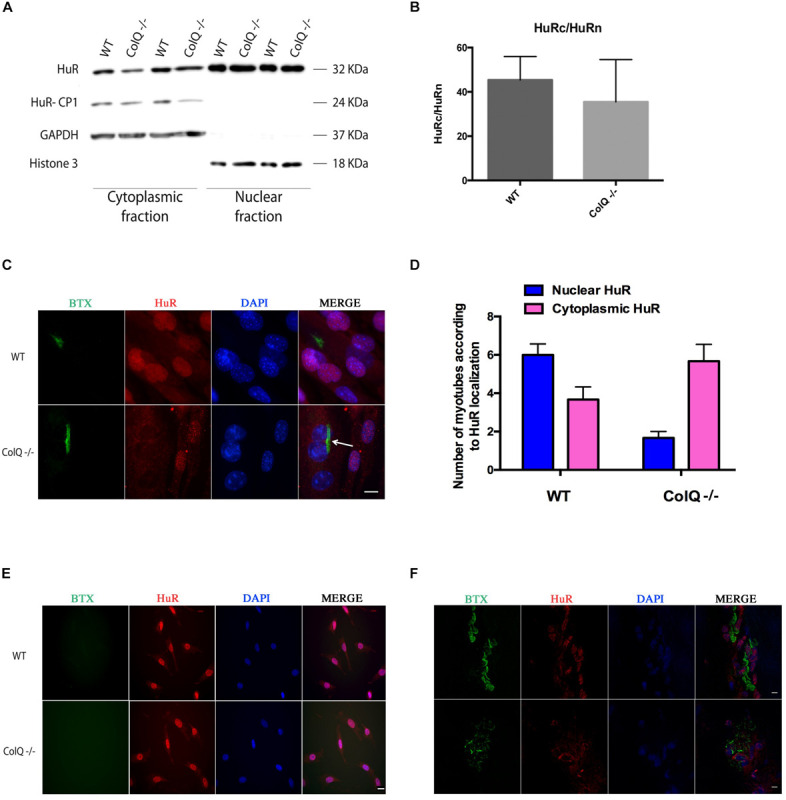
Immunostaining of HuR but not cell fractionation reveals its translocation to the cytoplasm in COLQ-deficient myotubes *in vitro* and *in vivo* **(A–E)**. **(A)** Representative western blot of myotube cell fractionation. GADPH was used as a cytoplasmic marker, histone 3 was used as a nuclear marker. **(B)** Quantification of the ratio between cytoplasmic HuR (HuRc) and nuclear HuR (HuRn) in WT and COLQ-deficient muscle cells. Mean values (*n* = 4 independent experiments) ± SEMs are shown. *p* < 0.05. **(C)** Immunostaining of HuR in WT and *ColQ* -/- myotubes. AChR clusters and HuR are visualized respectively with α-bungarotoxin (BTX) and HuR antibodies. Nuclei were stained with DAPI. **(D)** Quantification of AChR positive wt and ColQ-deficient myotubes with nuclear and cytoplasmic localization of HuR. Quantification was done in three independent experiments on a total of 29 wt myotubes and 22 ColQ-deficient myotubes. **(E)** Immunostaining of HuR in WT and *ColQ* -/- myoblasts performed as in panel **(C)**. **(F)** AChR, HuR and nuclei revealed as in panel **(C)** in adult mice muscle sections. Scale bar is 20 μm in panel **(C)**, 10 μm in panel **(E)** and 5 μm inpanel **(F)**.

In response to stimuli such as stress or in pathological conditions, HuR is known to translocate from the nucleus to the cytoplasm where it binds HuR mRNA targets as to increase their stability. We therefore quantified the levels of cytoplasmic (c) and nuclear (n) HuR at T2 in WT and COLQ-deficient muscle cells using cell fractionation. As expected, 2 forms of HuR protein are visualized in the western blot showing the cytoplasmic fraction (Figure 2A). These forms correspond to HuR full-length and a cleavage product of HuR termed HuR-CP1 that appears when HuR translocates to the cytoplasm. As shown in Figures 2A,B, HuR levels were rather variable in both cytoplasmic and nuclear fractions and no significant difference was observed for the HuRc/HuRn ratio between the 2 muscle cell lines (Figure 2B).

We performed immunofluorescence to visualize HuR in the 2 muscle cell lines at the same time point of cell differentiation, i.e., T2. HuR was observed in the nuclei of all WT and *ColQ* -/- myotubes without AChR clusters (pink nuclei in Figure 2C), whereas it was mostly located in the cytoplasm in *ColQ* -/- myotubes bearing AChR clusters (blue nuclei in Figure 2C, arrow) indicating that in *ColQ* -/- myotubes, HuR is translocated into the cytoplasm. Quantification of AChR positive myotubes performed on blue (cytoplasmic HuR) and pink (nuclear HuR) nuclei reveals that in wt myotubes, HuR is observed more often in nuclei than cytoplasm whereas in ColQ-deficient myotubes, HuR is predominantly present in the cytoplasm. The nucleo-cytoplasmic ratio was 1.63 in AChR positive wt myotubes and 0.29 in AChR positive ColQ-deficient myotubes. In these assays, myotubes labeled with AChR clusters represent a minor fraction of all the myotubes in both cell lines (around 1/12). This observation explains therefore the absence of a HuR difference between WT and COLQ-deficient muscle cells in the fractionation assay shown in Figures 2A,B, which was performed on total myotube lysates.

In myoblasts, where AChR clusters are not yet present in both cell lines, no difference was observed between WT and COLQ-deficient muscle cells in the localization of HuR which was predominantly nuclear (Figure 2D). Since all AChR transcripts (except AChR β-subunit mRNAs) are also upregulated *in vivo*, we hypothesized that similarly to cultured muscle cells, HuR localization could be affected by the absence of COLQ in mouse skeletal muscles. We thus visualized HuR in muscle sections of WT and COLQ-deficient adult mice. As shown on Figure 2E, HuR is mostly present in the nuclei of WT muscle fibers. Conversely, HuR accumulates in the cytoplasm of COLQ-deficient fibers suggesting that similar mechanisms of AChR subunit mRNA stabilization by HuR occur *in vivo* in absence of COLQ.

### HuR Mimics the AChR ß-Subunit mRNA Regulatory Effect Observed in Absence of ColQ

**FIGURE 3:**
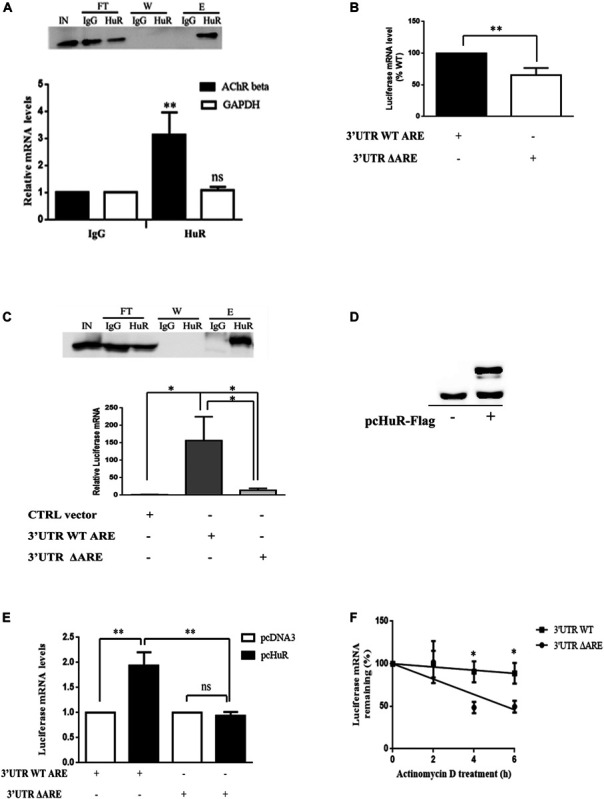
HuR binds endogenous AChR β subunit mRNA within the 3′UTR in myotubes. **(A)** Endogenous HuR interacts with AChR β-subunit mRNA *in vitro*. RNA–protein complexes were crosslinked with formaldehyde in WT differentiated myotubes and proteins immunoprecipitated with antibodies against HuR or IgG. A representative western blot in the top panel shows these proteins in the different fractions (IN, input: FT, flow through; W, wash; E, elution). Quantification of AChR β-subunit mRNA levels is shown below. Data are presented as mean percentages of 4 independent experiments. **p < 0.01; ns, not significant **(B)** Luciferase mRNA levels are decreased when 3′UTR ARE is mutated. Luciferase mRNA levels were quantified upon transfection of luciferase constructs containing the WT or mutated AChR β 3′UTR in COS cells (*n* = 4 independent experiments; **p < 0.01). **(C)** HuR regulates Luciferase mRNA stability via the ARE located in the AChR β 3′UTR. RIP assays were performed in COS cells transfected with WT-Luciferase or mutated 3′UTR Luciferase constructs (3′UTR WT ARE and 3′UTR ΔARE respectively) or the empty vector containing the Luciferase coding sequence without the AChR 3′UTR insert (CTRL vector) as a control. Top panel: representative western blot showing immunoprecipitated HuR (IN, input; FT, flow through; W, wash; E, Elution). Bottom panel: quantification of Luciferase mRNA levels (*n* = 4 independent experiments; *p < 0.05). **(D)** Western blot shows the expression of endogenous HuR and HuR-flag in transfected COS cells. **(E)** COS cells were co-transfected with Luciferase constructs containing the WT or mutated 3′UTR together with pcDNA3, or pcHuR-FLAG. Luciferase mRNAs were quantified by RT-QPCR and normalized to 18S (*n* = 4 independent experiments; **p < 0.01) **(F)** Luciferase mRNA stability assay in COS cells transfected with WT or mutated 3′UTR constructs. Luciferase mRNAs were quantified at each time point of actinomycin treatment and were normalized to 18S mRNAs. Values were expressed as a percentage of mRNA levels relative to time 0 (as in Figure 1A) and these values were plotted in a semi-log scale as in Figure 1A. *n* = 5 independent experiments. *p < 0.05; **p < 0.01.

To examine the potential link between the presence of HuR in the cytoplasm and the increased stability of AChR β-subunit transcripts in muscle cells, we tested a direct interaction between HuR and AChR β-subunit transcripts. We performed RNA immunoprecipitation (RIP) experiments in WT differentiated myotubes (at stage T2) grown in culture. After formaldehyde cross-linking and HuR or IgG (negative control) immunoprecipitation, co-immunoprecipitated mRNAs were analyzed by quantitative RT-QPCR (Figure 3A). Our results show that in myogenic cells, HuR interacts with AChR β-subunit mRNAs containing an ARE site and not with the glyceraldehyde 3-phosphate dehydrogenase (GAPDH) mRNAs that do not contain ARE sites.

In order to evaluate the contribution of the 3′UTR ARE in the AChR mRNA/HuR interaction, we used constructs previously described (Joassard et al., 2015). In these constructs, both a WT and an ARE mutated form of the full-length 3′UTR of the mouse AChR β-subunit transcript are inserted downstream of the luciferase reporter gene (3′UTR WT ARE and 3′UTR ΔARE, respectively) driven by a constitutive promoter. The mutated clone contains 3 point mutations in the ARE which prevents it from recruiting HuR. If the loss of COLQ is indeed regulating AChR β-subunit mRNA through HuR, overexpression of HuR should mimic the increased AChR mRNA stability observed in the absence of COLQ.

First, to test the role of HuR binding sites contained in the 3′UTR of AChR β-subunit, COS cells were transfected with 3′UTR WT ARE or 3′UTR ΔARE constructs. Cells were harvested 48 h after transfection and the Luciferase reporter transcript levels were measured by RT-QPCR (Figure 3B). The mutation in the ARE motif led to a significant 37% decrease in reporter transcript levels compared to the WT construct suggesting that endogenous HuR present in COS cells is not able to stabilize mutated transcripts containing a mutated ARE. Then, to show that HuR directly controls the levels of Luciferase transcripts through ARE, we transfected the ARE WT and ARE mutated Luciferase constructs into COS cells and performed a RIP assay using HuR antibodies. The RIP analysis, completed 48 h after transfection, showed a significant interaction of the 3′UTR WT construct with HuR when compared to that of the empty vector (Figure 3C, top panel for the western blot, bottom panel for the quantification of luciferase mRNA). Mutation of the conserved ARE (3′UTR ΔARE) resulted in a significant 13-fold decrease in the binding of HuR to the AChR β-subunit full-length 3′UTR, with measured abundance of mutant reporter mRNA in the immunoprecipitates being similar to that of the empty vector. These results confirmed that HuR interacts with the conserved ARE element in the 3′UTR of AChR β-subunit mRNA.

To further examine the impact of HuR on AChR β-subunit mRNA levels, we overexpressed HuR in COS cells using a HuR-Flag construct co-transfected with the 3′UTR WT ARE construct. As shown on the western blot on Figure 3D, the HuR-Flag construct was indeed overexpressed in addition to endogenous HuR, In these conditions, Luciferase mRNA levels were increased by 100% when compared to pcDNA3 empty vector (Figure 3E). The ARE mutant reporter construct, on the other hand presented no alteration in its mRNA levels even in conditions where HuR was overexpressed (Figure 3E).

To complement these experiments, we also compared the mRNA stability of the two constructs transfected into COS cells. To this end, 48 h post-transfection, we inhibited transcription by adding actinomycin D and then measured the relative abundance of luciferase mRNAs at 0, 2, 4, and 6 h thereafter (Figure 3F). As shown in Figure 3F, the degradation rate of the mutant ARE luciferase mRNA was significantly higher when compared to that of the control. Indeed, the half life of the mutant ARE luciferase mRNA calculated from the linear regression was 4.9 h whereas the half life of the wt ARE luciferase mRNA was 22.5 h. Taken together these results indicate that HuR binds the ARE element located within the 3′UTR of AChR β, stabilizes the transcript and, as a result, increases AChR β mRNA levels as found in ColQ-deficient cells.

### COLQ Regulates AChR β-Subunit mRNA Levels Through p38 MAPK Activity

**FIGURE 4:**
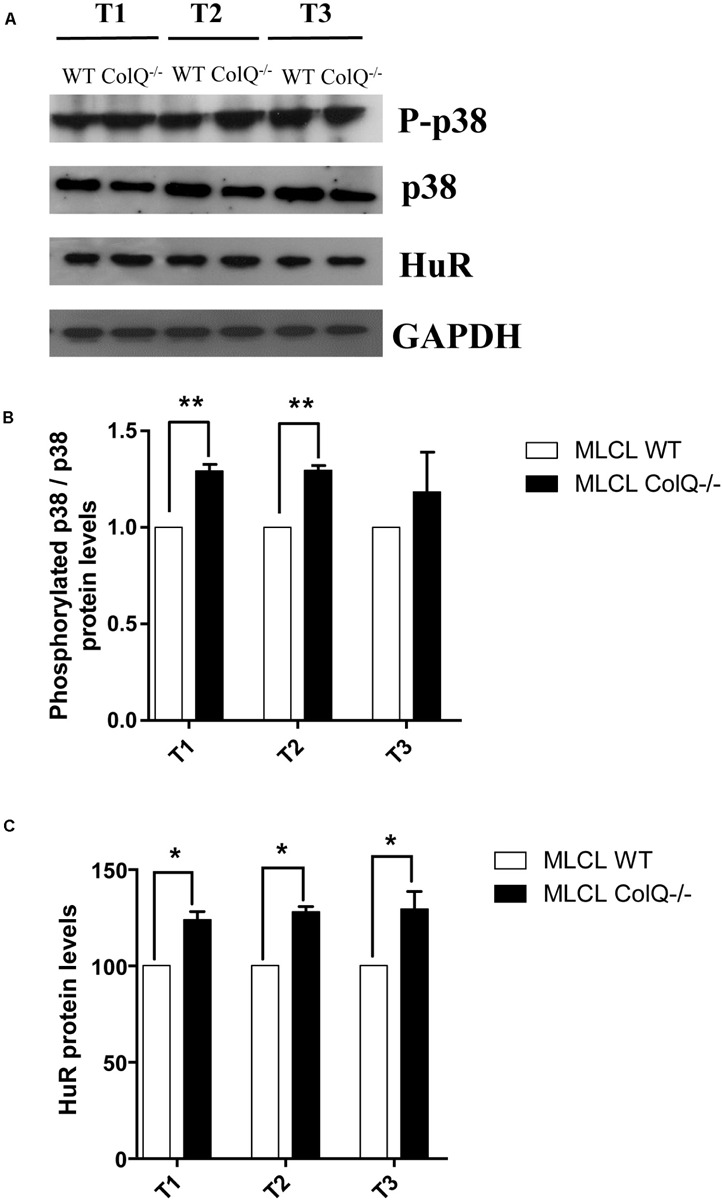
COLQ deficiency regulates p38 phosphorylation and HuR protein levels in myotubes. **(A)** Representative western blot showing the levels of phosphorylated p38 (P-p38), p38, HuR and GADPH in WT and *ColQ* -/- myotubes at different stages of differentiation from T1 to T3. **(B)** Quantification of the ratio phosphorylated-p38/p38. **(C)** quantification of HuR protein levels. P-p38, p38 and HuR levels were normalized to GADPH. *n* = 5 independent experiments. *p < 0.05; **p < 0.01.

The p38 MAPK pathway is known to play an important role in post-transcriptional regulation observed in ARE-containing mRNAs, by specifically regulating the abundance and/or activity of HuR (Srikantan and Gorospe, 2012). Furthermore, p38 activation has been shown to regulate AChR β-subunit mRNA stability in the mouse C2C12 muscle cell line (Joassard et al., 2015). Thus, to determine whether the absence of COLQ correlates with the activation of p38 signaling and HuR levels in differentiating muscle cells, we compared the phosphorylation levels of p38 in WT and COLQ-deficient cells at different stages of myotubes differentiation using western blots (Figure 4A). We quantified in parallel the ratios of phospho-p38/p38 and the levels of HuR in myotubes at T1, T2 and T3 (Figures 4B,C). The ratio of phospho-p38/p38 and the levels of HuR were both significantly higher in myotubes at T1 and T2 in absence of COLQ (Figures 4A–C) as well as the levels of HuR but not the ratio phospho-p38/p38 in these conditions at T3. It should be noted that although the levels of p38 seemed lower at the 3 time points in COLQ-deficient muscle cells compared to WT muscle cells, it remains that the levels of phosphorylated p38 were always higher in these cells. Thus, in myotubes, a concomitant upregulation of p38 phosphorylation and HuR levels occurs during muscle cell differentiation.

**FIGURE 5:**
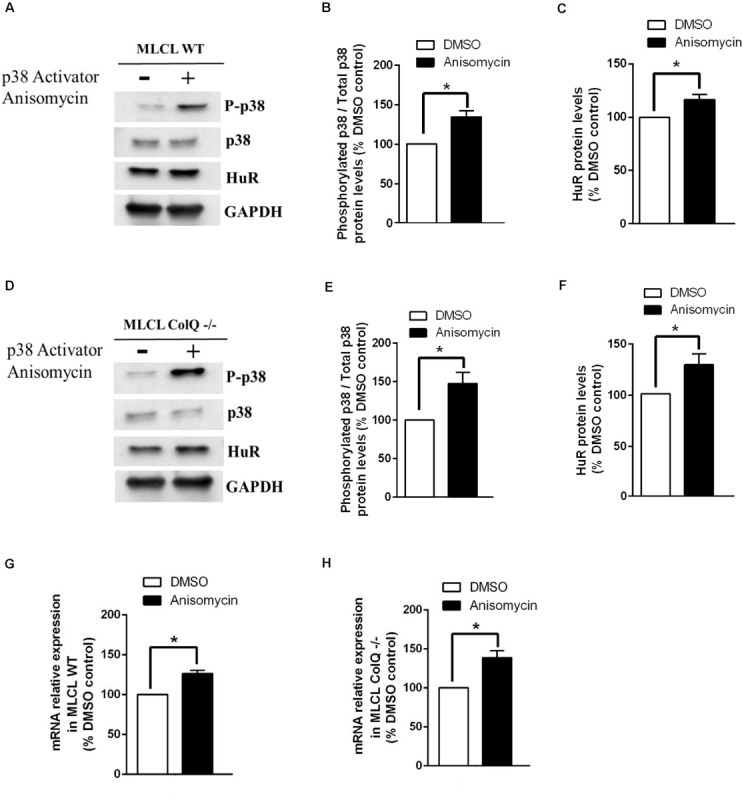
The treatment of WT or ColQ-deficient muscle cells with an activator of p38 induces parallel increases in p38 phosphorylation, HuR protein and AChR β-subunit mRNA levels **(A–H)**. **(A,D)** Representative western blots showing phosphorylated p38, p38, HuR, and GADPH in WT and *ColQ* -/- cells. **(B,C,E,F)** Quantification of these proteins in WT **(B,C)** and *ColQ* -/- cells **(E,F)**. **(G,H)** Quantification of AChR β-subunit mRNA levels. Quantitative data correspond to the means ± SEMs (*n* = 5 independent experiments). *p < 0.05; **p < 0.01.

**FIGURE 6:**
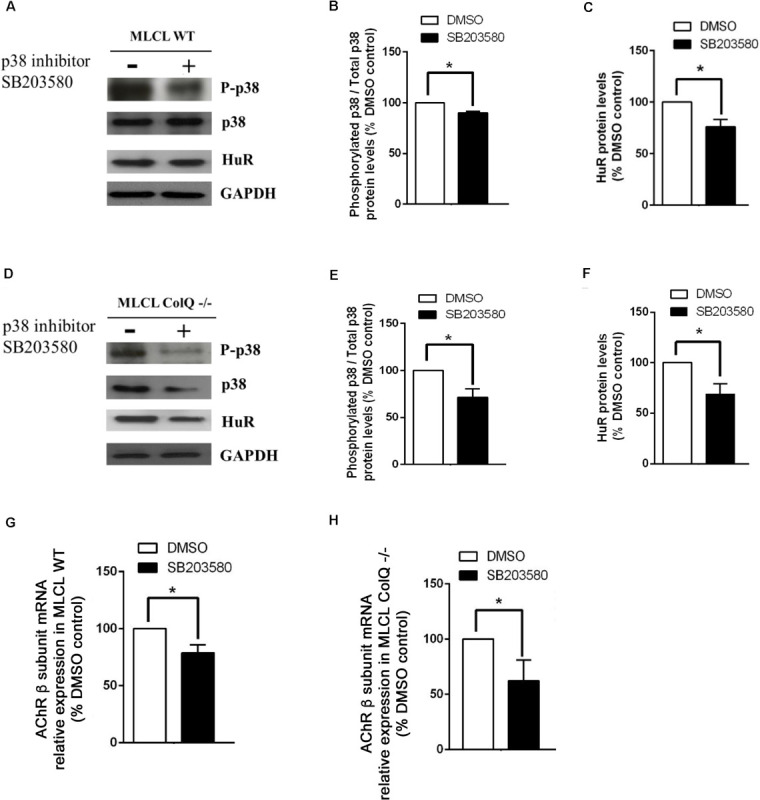
Inhibiting p38 phosphorylation with SB203580 induces parallel decreases in p38 phosphorylation, HuR protein and AChR β mRNA levels in WT muscle cell line **(A–H)**. Legends of the different panels are as in Figure 5. For quantifications, the means ± SEMs are shown (*n* = 5 independent experiments). *p < 0.05; **p < 0.01.

To further explore the relationship between p38, HuR and AChR β mRNA, we manipulated p38 activity by treating WT and COLQ-deficient myotubes at T2 with drugs that stimulate (anisomycin) or inhibit (SB203580) p38 activity, and quantified simultaneously the levels of phospho-p38/p38, HuR and AChR β-subunit mRNA. Analyses of western blots revealed that anisomycin-treated myotubes were responsive in both cell lines. At this stage, anisomycin treatment induced an increase in the ratio phospho-p38/total p38, HuR and AChR β-subunit mRNA in WT (Figures 5A,B,C,G) and *ColQ*-/- cells (Figures 5D,E,F,H). The 3 parameters were also affected by the p38α/β inhibitor SB203580 in WT cells (Figure 6). Western blot showed that inhibition of p38 activity resulted in a decrease in phospho-p38/total p38, in HuR protein levels and in AChR β-subunit mRNA levels in WT muscle cells (Figures 6A,B,C,G) as well as in ColQ-/- cells (Figures 6D,E,F,H). Altogether, in differentiated myotubes, HuR and AChR β mRNA levels are responsive to p38 activity.

## Discussion

The absence of COLQ or its low expression in the synaptic space at the NMJ is causative for a myasthenic syndrome with AChE deficiency. This neuromuscular disorder is mostly a consequence of high levels of ACh in the synaptic cleft due to the lack of AChE. As shown previously by our group, a striking effect of the absence of COLQ is an upregulation of all AChR subunit mRNAs correlated with an increase in the corresponding protein levels and the number of AChR clusters *in vivo* and *in vitro* (Sigoillot et al., 2010, 2016). Interestingly, other synaptic mRNAs and proteins in AChR clustering pathway such as MuSK and Rapsyn are also upregulated in the same condition (Sigoillot et al., 2016). This process is interpreted as an adaptation of the muscle cells to the overload of ACh in the synaptic cleft in an attempt to rescue the NMJ structure and function. Here, we show that a post-transcriptional mechanism of AChR mRNA stabilization is responsible for the increased levels of AChR β-subunit mRNAs observed in differentiated myotubes in response to COLQ deficiency. This mRNA stabilization is correlated with an increase in mRNA and protein levels of HuR and a cytoplasmic HuR enrichment in COLQ-deficient muscle cells. In addition, we provide evidence that ColQ deficiency stimulates p38 phosphorylation which in turn controls the levels of HuR and AChR β-subunit mRNA levels, a process that is specific of differentiated myotubes.

Until recently, the transcriptional regulation of AChR mRNAs induced by neurotrophic factors such as agrin and neuregulin, was described as the major mechanism responsible for the steady-state levels of these transcripts in skeletal muscle cells. These synaptic molecules induce the MAPK kinase (ERK), the PI3K and the JNK signaling pathways (Tansey et al., 1996; Lacazette et al., 2003; Burden et al., 2018), which converge on the activation of the Ets transcription factor, GABP (Schaeffer et al., 2001). Another member of the Ets family Erm has also been involved in the regulation of subsynaptic gene expression *in vivo* (Hippenmeyer et al., 2007) but only GABP has been shown to bind an N-box in the AChR subunit promoters inducing their transcription in synaptic nuclei (Schaeffer et al., 1998). Here, and in a previous study (Joassard et al., 2015), we found that the ARE present in the 3′UTR of AChR β-subunit transcript is a target of the RNA-stabilizing protein HuR. AREs are found in 4 of the 5 transcripts encoding AChR subunits, i.e., the α, β, δ, and γ transcripts^1^ and indeed we also found a strong interaction between HuR and the 3′UTR of AChR γ and α mRNA (data not shown). No canonical ARE could be detected in AChR ε transcripts but aside from the ARE described as the traditional HuR binding site motif, other U-rich or undetermined elements have been shown to bind this protein (Lopez de Silanes et al., 2004; Lebedeva et al., 2011) and could be responsible for the interaction between HuR and this AChR transcript.

Human antigen R is predominantly nuclear and translocate to the cytoplasm in response to stimuli to stabilize ARE- containing mRNAs. This is what occurs in COLQ-deficient myotubes where HuR is translocated to the cytoplasm in differentiated myotubes, a process that correlates with AChR β-subunit transcript increased stability. Interestingly, HuR’s cytoplasmic localization is only observed in COLQ-deficient myotubes bearing AChR clusters. We previously showed that the same percentage of myotubes showing AChR clusters is found in both WT and COlQ-deficient cell lines and that COLQ-deficient myotubes show a greater number of AChR clusters per myotube than WT myotubes (Sigoillot et al., 2010). This suggests that HuR could contribute to regulate AChR clustering through the stabilization of AChR mRNAs in COlQ deficient differentiated myotubes.

Our data indicate that COLQ-deficiency affects not only HuR localization but also HuR levels. This last process seems to be correlated to the level of p38 MAPK activity since activation or inhibition of this pathway correlates with increases or decreases of HuR levels, respectively. The level of HuR is known to be controlled by the transcription factor NF-κB that binds directly to the HuR promoter and by the TGFβ/Smad pathway (Kang et al., 2008; Jeyaraj et al., 2010). Post-transcriptional mechanisms also account for the regulation of HuR mRNA and protein levels since HuR itself contains ARE (Al-Ahmadi et al., 2009). Direct phosphorylation of HuR by p38α has been involved in HuR shuttling (Lafarga et al., 2009), but there is no evidence that p38 directly regulates HuR levels. However, p38α isoform has been found to activate NF-κB (Olson et al., 2007) which could in turn stimulate HuR expression. Thus the mechanisms by which p38 activation control HuR levels remains to be elucidated.

One key issue would be to identify the signaling pathway upstream of p38 activation. MAPK kinases including MKK3/6 are known to phosphorylate p38 MAPK. After skeletal muscle denervation, we previously showed that the activity of these 2 MKKs is stimulated which could be correlated to the enhanced activity of p38 in the same conditions (Joassard et al., 2015). Moreover, in our earlier study, we demonstrated that constitutively active mutant MKK6 transfected in C2C12 cells induces an increase in p38 activity and a parallel increase in AChR β-subunit mRNA levels and stability. It is thus probable that the same pathway is activated in our CMS model.

Since COLQ binds MuSK and its absence induces p38-HuR-mediated stabilization of AChR β mRNAs, one could hypothetize that MuSK is involved in p38 activation. A number of kinases have been shown to be activated by MuSK (Wu et al., 2010) but so far, there is no evidence that p38 can be recruited and activated by MuSK. In this context, the impact of COLQ on MuSK activity remains to be established. An alternative hypothesis is that p38 is activated by a COLQ-dependent indirect mechanism. In this last hypothesis, it should be reminded that one of the hallmarks of COLQ-deficient NMJ is the perturbation of the extracellular matrix organization that is illustrated by the presence of Schwann cells processes intruding into the synaptic cleft (Feng et al., 1999). This modification may induce a cellular stress and p38 has been reported as a major stress response-induced signaling pathway. The stress response could be mediated by postsynaptic transmembrane receptors such as integrins, which link the extracellular matrix to the cytoskeleton and are major sensor of mechanical forces in muscle. In this context, it will be interesting to explore the activity of the p38-HuR pathway in other myasthenic syndromes where the extracellular matrix (ECM) is affected, with particular focus on the synaptic class of these diseases such as the laminin β2-induced myasthenic syndrome. Indeed, similar morphological defects have been observed in laminin β2-mutants (Patton et al., 1998). Here, it should point out that several other components of the ECM such as perlecan and biglycan bind indirectly or directly to MuSK (Peng et al., 1999; Amenta et al., 2012) and thus MuSK could also act as a “sensor” of ECM integrity. Interestingly, in mice deficient for collagen VI that present NMJ defects, AChR mRNAs are also upregulated raising the question in this model of the mechanism used to regulate these transcripts (Cescon et al., 2018).

A similar scenario where HuR and p38 pathway are involved in mRNA stabilization in the context of neuromuscular disease is found in spinal muscle atrophy (SMA; Farooq et al., 2009, 2013). This disease is characterized by the progressive loss of motor neurons and consequently muscle atrophy. An early hallmark in this disease is the disruption of the NMJ (Kariya et al., 2008). The causative gene for the disease, survival motor neuron (SMN), has been identified (Lefebvre et al., 1995). In human, there are two similar genes coding for *SMN, SMN1*, and *SMN2.* Most of the patients lack SMN1 gene whereas SMN2 gene is expressed at low levels. Celecoxib, an activator of p38, has been proved to increase the levels of SMN proteins by activating the p38-HuR pathway in human and mouse neuronal cells (Farooq et al., 2013). The same p38-HuR pathway has been shown to stabilize AChR β transcripts in skeletal muscle denervation and we now demonstrate that this pathway is also activated in a model of myasthenic syndrome with AChE deficiency. In conclusion, our study extends the list of neuromuscular pathological situations where the p38-HuR pathway is activated to partially compensate for synaptic defects.

## Materials and Methods

### RNA Extraction and RT-PCR

Total RNA was extracted from COS-7 and muscle cells using TRIzol reagent (Invitrogen) as recommended by the manufacturer. TRIzol extracted RNAs were treated for 30 min at 37°C with DNAse followed by 10 min at 65°C with DNAse STOP (Promega kit) to eliminate possible DNA contamination. cDNAs were synthesized from DNase-treated RNAs using the reverse transcriptase (MuLV; Life technology). Real-time quantitative PCR was performed in triplicates on a 384-well plate with the ABI Prism 7900HT Sequence Detection System (Applied Biosystems). Using a QuantiTect SYBR Green PCR kit (QIAGEN, Valencia, CA, United States) according to the manufactures instructions, amplifications were performed with the following primer sequences: AChR β forward, 5’-CCTGAGGCCGCTATCCGGAC-3’ reverse, 5’-TTGGCCTTCGGCTTCCGACC-3’; Luciferase forward, 5’-AAACCATGACCGAGAAGGAG-3’ reverse, 5’-GTCCACGAACACAACACCAC-3’ GAPDH QuantiTect primer was purchased (Qiagen).

### RNA Immunoprecipitation

Muscle cells or COS-7 cells were cross-linked with 1% formaldehyde in PBS for 10 min at room temperature. The reaction was stopped with a wash in ice-cold PBS and resuspended in RIPA buffer. Cross-linked complexes were solubilized in a sonicator bath by six 30-s sonication pulses, and cellular debris were removed by centrifugation (14,000 *g* for 10 min at 4°C). Equal amounts of whole cell extracts were precleared with a Protein A/G plus beads (Santa Cruz) previously blocked with 200 μg/ml of competitor tRNA, and 40 μg/ml of salmon sperm DNA. Complexes were immunoprecipitated using 3 μg of of HuR (Santa Cruz 3A2; 1:500) or IgG antibodies (Sigma M5284; DIL?) and protein A/G plus beads overnight at 4°C. After three washes with RIPA buffer and two washes with TE buffer (10 mM Tris–HCl, pH 8.0, and 1 mM EDTA), beads containing the immunoprecipitated samples were resuspended in 100 μl of elution buffer (50 mM Tris-HCl, pH 7.0, 5 mM EDTA, 10 mM DTT, and 1% SDS). Cross-linking was reversed at 70°C for 5 h. Samples were taken throughout the protocol for RNA extraction and further RT-QPCR and western blotting to confirm the efficiency of immunoprecipitation.

### Mice, Muscle Cell Lines, Culture, Treatments, Transfection, Immunohistochemistry and Cell Fractionation

Experiments using ColQ-deficient mice were performed in compliance with the European Community guidelines (N°A-75-1970). The generation of the wild type (WT), ColQ-deficient (ColQ-/-) muscle mMLCL cell lines has been previously described in Sigoillot et al. (2010). In the abbreviation mMLCL, “m” stands for mouse and “MLCL” are the initials of the researchers who made the cell lines. Myoblasts were maintained in growth medium supplemented with 10% fetal bovine serum, 20% horse serum, 2 mM glutamine, 2% penicillin/streptomycin (5000 U) and 20 U/ml γ-interferon (Roche Diagnostics) at 33°C in 8% CO_2_. Myoblasts were induced to differentiate into myotubes on collagen type I coated plates in medium supplemented with 5% horse serum, 2 mM glutamine, and 2% penicillin/streptomycin (5000 U). WT and ColQ-deficient cells were treated with a p38 activator anisomycine (100 nM, 1h; Sigma-Aldrich) or a p38 inhibitor SB203580 (10 μM, 1h; Sigma-Aldrich). SB203580 inhibits p38α and p38ß but not p38γ. COS cells were transfected using Fugene HD (Promega), according to the manufacturer’s instructions and resulted in <70% of cells expressing the transfected cDNAs. AChR clusters and HuR were visualized respectively with α-bungarotoxin alexa fluor 488 conjugate (1:500; Fisher) and a monoclonal antibody against HuR (1:100, 3A2; Santa Cruz) revealed with a goat anti-mouse alexa fluor 594 (1:500; Thermo Fisher Scientific). The same toxin and antibodies were used to stain AChR and HuR on sections of muscles from adult wt and ColQ-deficient mice. Cell fractionation was performed using the nuclear/cytosol fractionation kit (BioVision) and according to the protocol provided by the manufacturer.

### Western Blot Analysis

Proteins were extracted from cell lines using a lysis buffer containing: TrisHCl 50 mM, NaCl 150 mM, EDTA 2mM, Sodium orthovanadate 2 mM and TritonX100 1%. Total proteins were isolated from cell debris by centrifugation. Equal amounts of total protein (30 μg/sample) were separated on a 12% SDS-PAGE Gel and transferred onto nitrocellulose membranes. Non-specific binding was blocked with PBS and 0.1% Tween containing 5% skim milk for non-phosphorylated proteins and TBS 0.1% Tween containing 3-5% BSA when analyzing phorsphorylation state. Membranes were then incubated with primary antibodies for 1 h at RT or overnight at 4°C. After washing with PBS and 0.1% Tween or TBS 0.1% Tween (phosphorylated proteins), membranes were incubated with HRP-conjugated secondary antibodies. Repated washing was followed by revelation using ECL detection reagents (GE Healthcare) and visualized using hyperfilm ECL or ImageQuant LAS 4000. Quantifications were performed with ImageJ software. The antibodies were the following: a mouse monoclonal anti-GAPDH clone 6C5 (1: 10000; Abcam), a mouse monoclonal anti-HuR 3A2 (1:500; Santa Cruz), an anti-mouse IgG HRP (1:10000; Sigma), an anti-rabbit IgG HRP (1:10000; Sigma), an anti-histone 3 (1:1000; Cell Signaling Technologies), a mouse monoclonal anti-phosphorylated p38 (1:2000, clone 28B10; Cell Signaling Technology) and a rabbit polyclonal anti-p38 (1:1000; Cell Signaling Technology).

### Statistical Analysis

Unpaired Mann–Whitney’s *U* tests were used to determine the significativity between different groups of data. All quantifications are expressed as means ± SEM.

## Data Availability Statement

The raw data supporting the conclusions of this article will be made available by the authors, without undue reservation.

## Ethics Statement

Ethical review and approval was not required for the animal study because cell culture.

## Author Contributions

JK, PD, FS, NS, JA, GB, AR-C, and AD did the experiments. BJ and CL designated the experiments. CL coordinated and supervised the experiments. All authors contributed to the article and approved the submitted version.

## Conflict of Interest

The authors declare that the research was conducted in the absence of any commercial or financial relationships that could be construed as a potential conflict of interest.

## Footnotes

1 http://brp.kfshrc.edu.sa/ared

## Abbreviations

ACh: acetylcholine
AChR: acetylcholine receptor
ARE: AU-rich element
BTX: α -bungarotoxin
COLQ: collagen Q
Dok7: docking protein a
Elavl1: embryonic lethal abnormal vision like 1
ECM: extracellular matrix
GABP: GA-binding protein
GAPDH: glyceraldehyde 3-phosphate dehydrogenase
HuR: human antigen R (synonym of Elavl1)
JNK: c-jun NH2-terminal kinase
LRP4: low-density lipoprotein receptor-related protein 4
MAPK: mitogen-activated protein kinase
MyoD: myoblast determination protein
MuSK: muscle specific kinase
NF- κ B: nuclear factor kappa-light-chain-enhancer of activated B cells
NMJ: neuromuscular junction
SMN: survival motor neuron
UTR: untranslated region.
